# Multi-Objective Optimisation of Curing Cycle of Thick Aramid Fibre/Epoxy Composite Laminates

**DOI:** 10.3390/polym13234070

**Published:** 2021-11-23

**Authors:** Guowei Zhang, Ling Luo, Ting Lin, Boming Zhang, He Wang, Yuao Qu, Bangke Meng

**Affiliations:** 1 School of Materials Science and Engineering, Beihang University, Beijing 100191, China; zbm@buaa.edu.cn (B.Z.); sy1901329@buaa.edu.cn (H.W.); 2 Aerospace Institute of Advanced Materials & Processing Technology, Beijing 100074, China; luoling_buaa@yeah.net; 3 Design and Development Center, AECC Commercial Aircraft Engine Co., Ltd., Shanghai 201104, China; z2250898783@gmail.com; 4 Dongxiaokou Community Health Service Center, Beijing 100192, China; quyuao@126.com; 5 Technology Department, JOY Composites Co., Ltd., Tai’an 271033, China; mbk@bjcomposites.com

**Keywords:** composite materials, aramid fibre/epoxy, thick laminate, overheating temperature, optimisation

## Abstract

Aramid fibre-reinforced epoxy composites (AF/EP) are promising materials in the aerospace, transportation, and civil fields owing to their high strength, high modulus, and light weight. Thick composite laminates are gradually being applied to large composite structures such as wind turbine blades. During curing, temperature overheating is a common problem in thick composites, which leads to matrix degradation, thermal residual stresses, and uneven curing. This paper proposes a signal-to-noise ratio (SNR) method to optimise the curing cycle of thick AF/EP laminates and reduce the overheating temperature. During curing, the temperature and strain evolution in a thick AF/EP laminate were monitored using fibre Bragg grating sensors. The effects of the curing factors on the overheating temperature of the thick AF/EP laminate were evaluated using the Taguchi method and predicted via the SNR method and analysis of variance. The results indicate that the dwelling temperature is the main factor affecting the overheating temperature. The optimal curing cycle involves an overheating temperature of 192.72 °C, which constitutes an error of 2.58% compared to the SNR method predictions. Additionally, in comparison to the initial curing cycle, the overshoot temperature in the optimised curing cycle was reduced by 58.48 °C, representing a reduction ratio of 23.28%.

## 1. Introduction

Owing to its high strength, high modulus, low density, impact resistance, high-temperature resistance, wear resistance, chemical corrosion resistance, fatigue resistance, and dimensional stability [1,2,3,4,5,6,7,8], fibre-reinforced plastic (FRP) has occupied a prominent position in the aerospace, aviation, and national defence industry. Aramid fibre-reinforced epoxy (AF/EP) is a high-performance composite material with aramid fibres as the reinforcement. With the increasing maturity of the AF/EP composite process technology, its application has gradually expanded from the initial aerospace and military fields to civil fields, such as in sporting goods [9,10]. The autoclave process is a common method for producing high-performance thermosetting resin matrix composites [11,12,13,14,15]. In the autoclave process, AF/EP prepregs are stacked according to specific layers and cured. The process conditions, stability of the resin content, and uniformity of the material properties directly affect the mechanical properties of AF/EP composites. To maximise the excellent properties of AF/EP composites, it is essential to determine the optimal curing cycle.

Thick composite laminates (≥6 mm) [16] are gradually being applied to large composite structures such as wind turbine blades. The conventional autoclave curing cycle recommended by the prepreg manufacturer is only used for the manufacturing of thin laminates; when thick laminates are cured under the same cycle, the low thermal conductivity of the EP matrix hinders heat release, causing the temperature at the centre of the thick laminate to increase instantaneously during the exothermic reaction [17,18]. This overheating temperature phenomenon leads to matrix degradation, thermal residual stresses, and uneven curing [19], therefore affecting the final mechanical properties of the composites [20]. Some scholars have studied the curing of thick composites. Cassano [21] proposed a methodical approach coupled with an analytical cure kinetics model in the commercial software Abaqus by user subroutines was to aid the design of cure cycles for thick glass fibre/EP laminates. Loos and Springer [22] conducted an experimental study on 64-ply thick graphite/EP laminates and developed a computer code to predict the temperature distribution in the laminates. They showed that the thick laminates can achieve a uniform temperature if the heating rate is sufficiently low. Hjellming and Walker [23] studied the curing cycle of 150 mm- and 300 mm-thick-wall graphite/EP composite cylindrical structures and showed that with an increase in the wall thickness, the curing cycle is prolonged, although the curing cycle duration and thickness do not follow a simple scaling law. Yang [24] developed a simulation method that can effectively reduce the temperature and curing degree gradients along the thickness of glass fibre/EP composite product. Guan [25] optimised the curing cycle for T800/X850 carbon fibre-reinforced plastic while ensuring mechanical properties. Muc [26] proposed a two-dimensional simulation method to discuss the influence of the resin curing process on residual stresses in thick thermosetting composites and concluded that by choosing an optimised curing cycle, the residual stresses could be reduced substantially. Lee [27] measured the temperature distribution in a 20 mm-thick unidirectional glass/EP laminate in the autoclave process and compared it with numerical results. They showed that for thin laminates, the curing temperature exhibits an insignificant gradient over the entire thickness range, whereas in thick laminates, a strong temperature gradient is observed. This gradient is related to the change in the curing cycle with thickness and it is more evident in thicker laminates. The temperature gradient is caused by the exothermic reaction of the curing process and thermal diffusion, depending on the composite laminate thickness and thermal conductivity. Thus, in existing literature, the overheating temperature in thick AF/EP laminates has not been reported.

The Taguchi method is an economical and efficient experimental design method. It emphasises optimisation through design and not through many experiments and introduces the influence of uncontrollable factors into the experimental design [28,29,30,31,32,33]. The Taguchi method combines orthogonal experiments and analysis of variance (ANOVA) [34,35,36,37,38,39] to determine the best combination of controllable factors to reduce the impact of uncontrollable factors on system quality fluctuations. Furthermore, the signal-to-noise ratio (SNR) method is an interdisciplinary evaluation and prediction approach [40,41]. To our knowledge, this is the first study to apply the SNR method to evaluate and predict the temperature overshoot phenomenon in thick composites.

This study aims to optimise the curing cycle of thick AF/EP laminates. First, to understand the curing process of thick AF/EP laminates, temperature and strain monitoring was performed using fibre Bragg grating (FBG) sensors, which, owing to their durability, reliability, and small size, do not weaken the structure [42,43,44]. Second, using the Taguchi method’s orthogonal experiment design during the temperature monitoring experiment, the influence of process factors on the overheating temperature was determined, and the SNR method was applied to evaluate the temperature. ANOVA was applied to determine the optimal curing cycle, and the SNR method was used to anticipate the overheating temperature under the optimal curing cycle. Finally, the accuracy of the SNR method was evaluated through experiments. This work provides a new method for reducing and predicting the overheating temperature.

## 2. Theoretical Approaches

### 2.1. Signal-to-Noise Ratio Method

The SNR was originally defined as the ratio of a normal sound signal to a signal-to-noise signal; it serves as a method to evaluate the quality of audio products. This research introduces the use of the SNR to evaluate and predict the overheating temperature of thick AF/EP laminates. The SNR conversion equation can be expressed as follows [41]:

(1) $$\mathrm{SNR}\;\left(\eta \right)=-10\log_{10}\left[\frac{1}{n}\overset{n}{\underset{i=1}{\sum }}\;\frac{1}{y_{i}^{2}}\right],$$

In Equation (1), *η* denotes the SNR (dB); *y_i_* denotes the overheating temperature value; and *n* denotes the number of experiments.

The SNR equation used for prediction is as follows:

(2) $$\eta_{p}=\eta_{m}+\overset{j}{\underset{i=1}{\sum }}\left(\eta_{f}-\eta_{m}\right),$$

where $\eta_{p}$ is the predicted SNR, $\eta_{m}$ is the mean SNR based on all experiments, $\eta_{f}$ is the SNR at the optimal level of a factor, and *j* is the number of factors.

First, the SNR value was calculated from the overheating temperature using Equation (1). Secondly, the optimal curing cycle was obtained by the ANOVA of the SNR. Equation (2) can calculate the predicted SNR under the optimal curing cycle. Finally, the predicted overheating temperature was calculated from the predicted SNR using Equation (1). The predicted overheating temperature was experimentally verified.

### 2.2. Fibre Bragg Grating Sensing Principle

**Figure 1:**
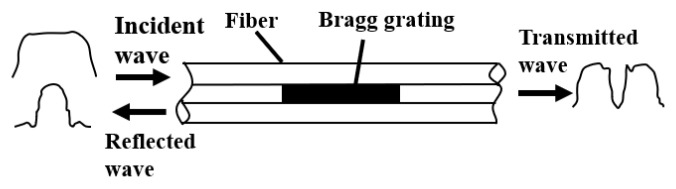
Working principle of fibre Bragg grating.

When light is transmitted through an FBG, the refractive index of the FBG is periodically disturbed, owing to the action of the temperature and force, to reflect light of a specific wavelength, which is termed as the Bragg wavelength (Figure 1).

The Bragg equation is as follows [45]:

(3) $$\lambda_{B}=2n_{\mathrm{eff}}\;\Lambda ,$$

where $\lambda_{B}$ is the reflected light wavelength, $n_{\mathrm{eff}\;}$ is the refractive index of the fibre core, and Λ is the Bragg period of the grating.

The sensor also exhibits a linear response to both strain and temperature, making it suitable for structure monitoring applications [46]. A change in temperature and strain will result in a change in the FBG wavelength, and the spectrum centre wavelength $\Delta \lambda_{B}$ is determined by

(4) $$\Delta \lambda_{B}=K_{\varepsilon }\varepsilon +K_{T}\;\Delta T,$$

where $K_{\varepsilon }$ is the strain sensitivity constant, $K_{T}$ is the temperature sensitivity constant, ***ε*** is the strain, and ΔT is the temperature increment. Both the constants are calibrated in this work.

## 3. Experimental

### 3.1. Calibration Experiment of Kε

**Figure 2:**
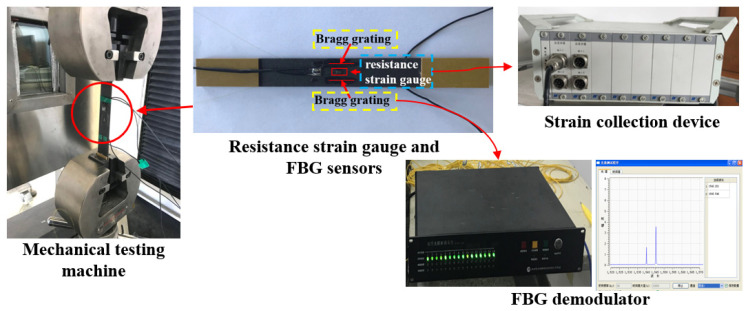
Resistance strain gauge and FBG sensors.

The diameter of the FBG sensors (procured from Yuguang Inc., Hangzhou, China) was 125 μm. One resistance strain gauge was welded to the centre of the tensile specimen along the tensile direction. Two FBG sensors were pasted on the specimen parallel to the strain gauge, as shown in Figure 2. The drawing speed during tensile testing was 2 mm/min.

### 3.2. Curing Monitoring Experiment

**Figure 3:**
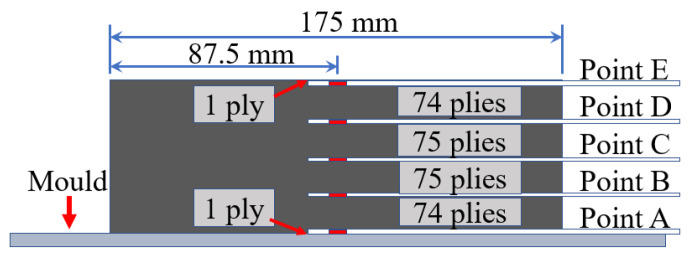
Location of five monitoring points.

The temperature sensitivity constant was estimated through the experiment. The material used in this work was the plain-woven AF/EP prepreg (procured from AVIC Composite Corporation LTD, Beijing, China). The size of the [0]_300_ thick AF/EP laminate was 175 mm × 80 mm, and it had a thickness of 51 mm (before curing). The thick AF/EP laminate was manufactured using the autoclave process. The autoclave curing cycle recommended by the prepreg manufacturer is as follows: the temperature is raised from 25 °C to 130 °C at a heating rate of 0.5 °C/min and then held constant for 120 min. After holding, the temperature is reduced from 130 °C to room temperature (25 °C) at a rate of 0.3 °C/min. During the entire autoclave process, the pressure was maintained at 6 bar. Five monitoring points (A, B, C, D, and E) in the AF/EP laminate, located along the thickness, were chosen, as shown in Figure 3.

**Figure 4:**
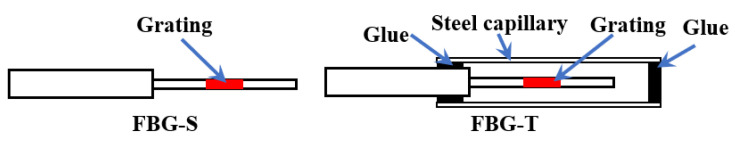
Two types of FBGs.

**Figure 5:**
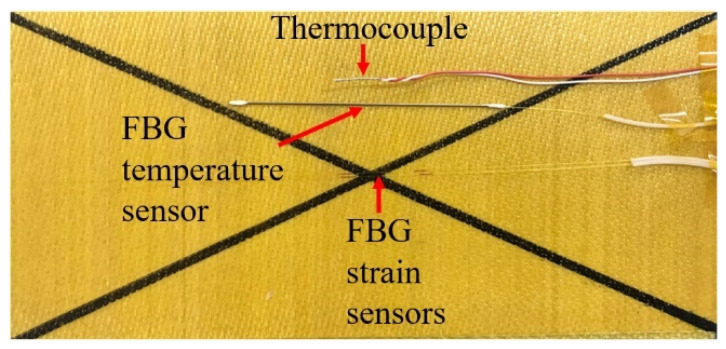
Distributions of FBGs and thermocouple.

**Figure 6:**
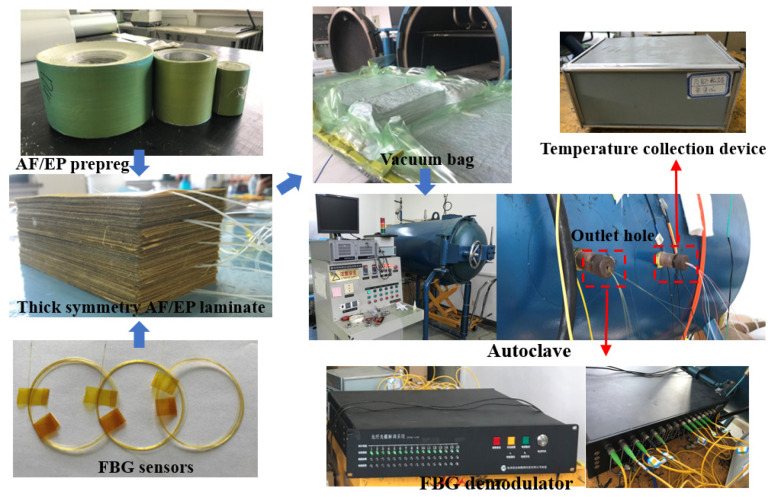
Curing monitoring experiment process.

Two types of FBGs were used in this study (Figure 4): FBGs affected by the combined action of temperature and strain (denoted as FBG-S) and FBGs affected by temperature alone (denoted as FBG-T). FBG-T was protected by a steel capillary tube from epoxy resin curing. One FBG temperature sensor and two FBG strain sensors were assigned to each monitoring point. Owing to the limitation of the outlet hole of the autoclave, only three thermocouples (produced in Shanghai E-B Automation Instrument CO., Ltd., Shanghai, China) were used, and these were embedded into points A, C, and E. Figure 5 shows the distributions of the FBG sensors and the thermocouple. The curing monitoring experiment process is shown in Figure 6.

### 3.3. Orthogonal Experimental Design

**Figure 7:**
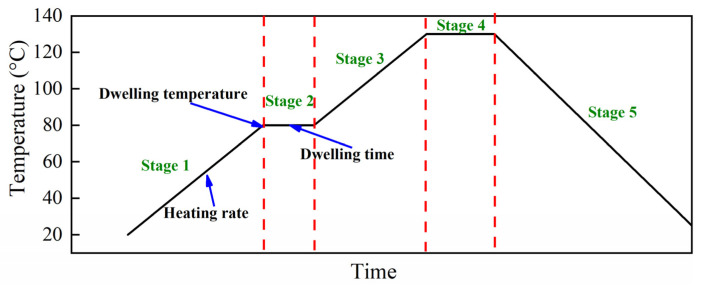
New curing cycle.

In this work, the overheating temperature phenomenon was monitoredTo reduce the overshoot temperature, a process whereby a new curing cycle is proposed. Temperature dwelling was added before the curing temperature (130 °C), as shown in Figure 7. The design principle of the new curing cycle is that the curing temperature, holding time, and cooling rate provided by the prepreg supplier remain unchanged; in other words, the curing temperature (130 °C) holding 120 min (stage 4) is unchanged, and the stage 5 that temperature is reduced from 130 °C to room temperature (25 °C) at a rate of 0.3 °C/min is also unchanged. Stage 2 involves the temperature dwelling added to the new curing cycle, and the temperature and holding time during this dwelling are referred to as the dwelling temperature and the dwelling time, respectively. The heating rates of stages 1 and 3 are the same; however, the durations of these two stages change with the heating rate and dwelling temperature. Thus, this work proposes three factors (heating rate, dwelling temperature, and dwelling time) affecting the process. Taguchi’s orthogonal array was used to design experiments to evaluate the influence of these three factors on the overheating temperature.

Taguchi’s method has many mature orthogonal arrays to operate experiments for investigating the influence of various factors. This is advantageous because it is an economical and efficient experimental design method. For the curing process of thick AF/EP laminates, three factors (heating rate, dwelling temperature, and dwelling time) set at four levels were studied to determine their effects on the overheating temperature. A three factor and four level orthogonal array was selected (L_16_), as presented in Table 1. Factor A was the heating rate, which was set to 0.25, 0.5, 0.75, and 1.0 °C/min; Factor B was the dwelling temperature, which was set to 80, 90, 100, and 110 °C; and Factor C was the dwelling time, which was set to 30, 60, 90, and 120 min. These details are listed in Table 2. The level combinations of factors and the obtained experimental results of the overheating temperature and SNR are shown in Table 3.

## 4. Results and Discussion

### 4.1. Strain and Temperature Sensitivity Constants

**Figure 8:**
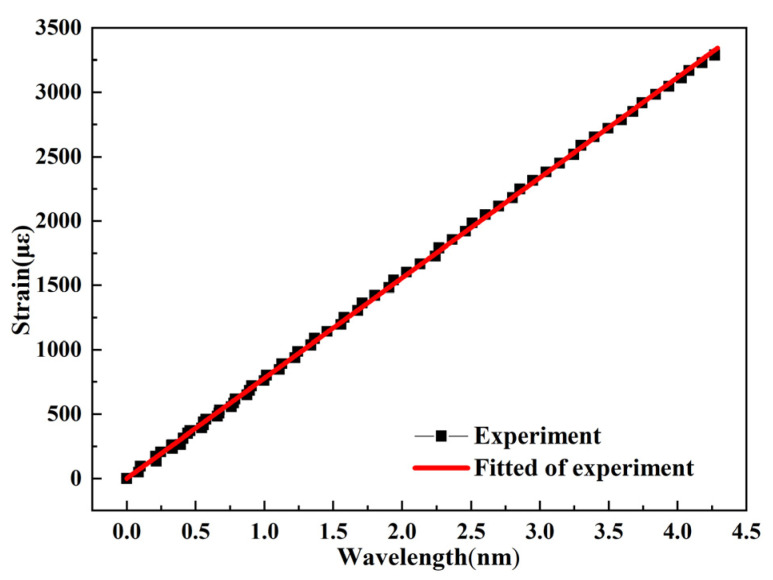
Relationship between strain and wavelength.

Figure 8 presents a strong linear relationship between the reflected wavelength from the FBG sensor and the strain from the strain gauge. The strain sensitivity constant (*Kε*) of the FBGs was 1.263 pm/με.

**Figure 9:**
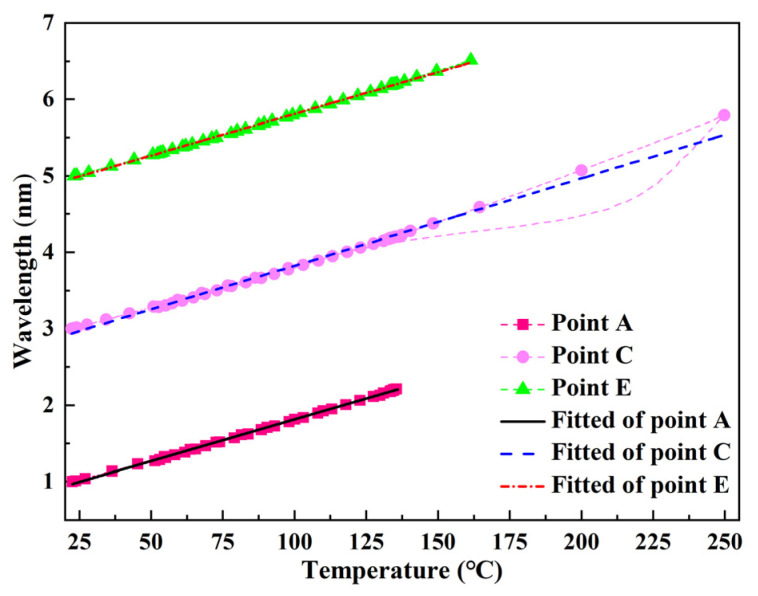
Relationship between temperature and wavelength.

**Figure 10:**
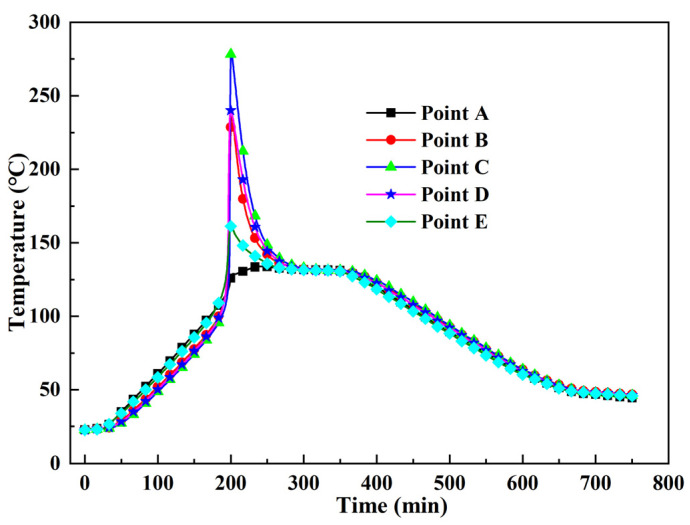
Temperature history of thick AF/EP composites.

Figure 9 shows the different reflected wavelengths from the FBG corresponding to the temperatures of the thermocouples at points A, C, and E. Three temperature sensitivity constants were acquired: *K_TA_* = 10.9 pm/°C, *K_TC_* = 11.4 pm/°C, and *K_TE_* = 10.9 pm/°C. Compared with the curves for points A and E, the linearity of the curve from point C was poor, especially near the overshoot temperature; further, the drastic exothermal reaction caused the temperature to increase sharply (as shown in Figure 10). Thus, the overheating temperature had a significant influence on the wavelength and temperature linearity.

### 4.2. Curing Process of Thick AF/EP Laminates

**Figure 11:**
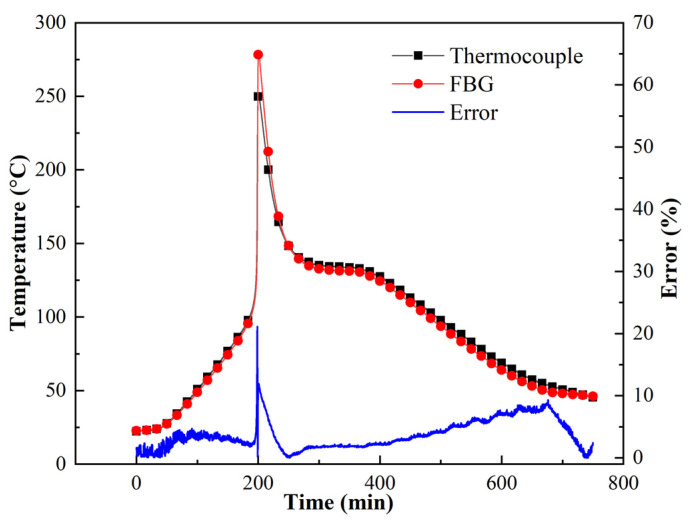
Comparison of temperatures monitored by FBG and thermocouple.

The temperature histories of the five monitoring points are shown in Figure 10. During the heating process, points A and E were heated faster than the inner points. On approaching the curing temperature, the temperatures at points B, C, D, and E increased rapidly owing to the severe curing reaction. The highest temperature of point C was 281.16 °C, which was also the highest temperature in the thick laminate because the highest temperature occurred at the middle point [17,18]. However, the temperature measured by the thermocouple was 251.2 °C (Figure 11). This error is related to the sharp increase in temperature. The maximum temperatures at points B and D were 235.73 °C and 240.12 °C, respectively, exhibiting little difference. Point A displayed a maximum temperature of 134.18 °C, which was only 4 °C higher than the curing temperature. Point E exhibited a maximum temperature of 161.35 °C. The temperature difference of 27 °C between points A and E was mainly because the heat dissipation performance of the metal mould was considerably better than that of the vacuum bag. The temperature distribution was evidently different in the curing process of the thick AF/EP composites.

Figure 11 shows the time–temperature curve at point C, as monitored by the thermocouple and FBG. The overshoot temperature monitored by the FBG was 281.16 °C, whereas the temperature monitored by the thermocouple was 251.2 °C. Thus, a temperature difference of 29.96 °C was noted between these readings. As shown in Figure 11, the measurement error of the FBG is less than 5% before the occurrence of the overshoot temperature and 21.16% immediately after the occurrence of the overshoot temperature; the maximum error in the cooling stage is 11.18%. Therefore, the overheating temperature has a significant impact on the accuracy of FBGs. Consequently, it is necessary to control and reduce the overheating temperature. Importantly, this is the first study to show that the overheating temperature during the curing process of composites affects FBG sensor measurements.

**Figure 12:**
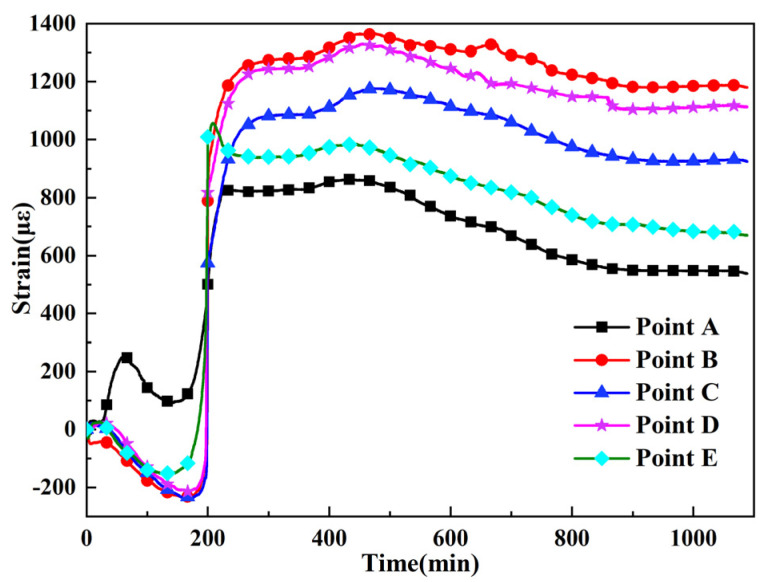
Strain history of thick AF/EP composites.

Figure 12 illustrates the strain history of the five points throughout the curing process. This is a complex process influenced by the temperature, curing shrinkage, and mould interaction. Before the gelation of the resin, the strain changes caused by thermal deformation and the resin flow of the prepreg were measured using the FBG sensors. In the initial stage, the trend of the strain at point A was different from those of the strains at the other points; this was likely due to the thermal expansion of the mould. At around 200 min, the strains at all the five points increased rapidly within a very short period. Before the gelation of the resin, thermal expansion and solidification do not result in strains [43]. However, the strain curves increased primarily due to the excessively large thermal expansion caused by the heat from the drastic exothermal reaction and the frictional effect transferred to the FBGs as the viscosity increased. In the thermal insulation platform stage, the resin reacted until it was completely solidified. The curing shrinkage can only be clearly observed in the strain curve for point E. Therefore, the heat of the drastic exothermal reaction was the most important factor affecting the strain of the thick AF/EP composites. In the cooling stage, the strain curves decreased manly due to the reduction in temperature and the transfer of the frictional effect to the FBGs. The minimum curing residual strain at point A, which was affected by the mould, was 537.66 με. The residual strain at point E was 669.10 με due to the direct effect of the atmospheric pressure of 6 bar acting on the outer layer, which limited the strain development during the forming process. The residual strain values at points B, C, and D were 1180.63 με, 925.66 με, and 1114.12 με, respectively. The strain gradient distribution was notably different in the curing process of thick AF/EP composites.

### 4.3. Evaluation of Process Parameters

During the curing of the thick AG/EP laminate, the overheating temperature leads to matrix degradation, thermal residual stress, and uneven curing [19]. Hence, a lower overheating temperature was pursued during curing. Table 4 shows the ANOVA of the SNR; the dwelling temperature was found to be the primary factor with a contribution of 75.78%. The contributions of the heating rate and dwelling time were 20.16% and 4.06%, respectively.

**Figure 13:**
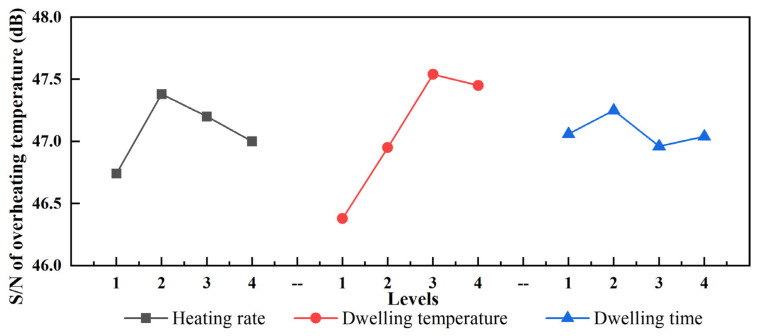
Mean SNR values under four levels of three factors.

The mean SNR values under four levels of the three factors are shown in Figure 13. The heating rate, dwelling temperature, and dwelling time simultaneously determine the overheating temperature caused by the curing reaction. The combination of the best levels of the curing factors was A1B1C3; in other words, a lower overheating temperature was obtained at a heating rate of 0.25 °C/min, dwelling temperature of 80 °C, and dwelling time of 90 min. The overheating temperature of the thick AF/EP laminate under the best curing cycle was 197.70 °C, calculated by Equations (1) and (2). The optimal curing cycle was predicted, but it is not in Table 3. Therefore, further experiments should be carried out to validate the predicted curing cycle.

**Figure 14:**
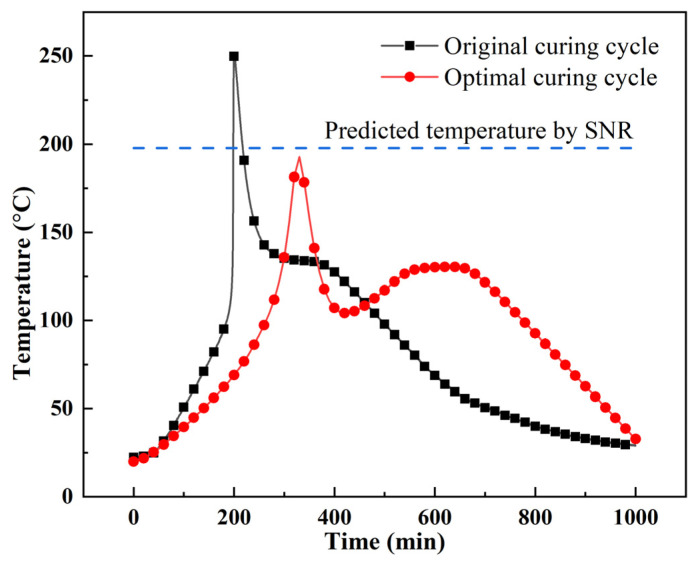
Comparison of experimental and prediction results.

The time–temperature relationship of the thick AF/EP laminate under the original and optimal curing cycle is shown in Figure 14. In the experiment, the overheating temperature of the thick AF/EP laminate was 192.72 °C; it involved an error of 2.58%, as compared with the overheating temperature (197.70 °C) predicted using the SNR method. Moreover, compared with the curing cycle provided by the prepreg manufacturer, the overheating temperature was reduced by 58.48 °C, representing a reduction ratio of 23.28%.

## 5. Conclusions

This paper proposed an optimisation method for the overheating temperature phenomenon in [0]_300_ thick AF/EP laminates, and the effects of the curing factors were discussed. The conclusions drawn are as follows:

1. FBG sensors can be used to monitor the temperature and strain produced during the curing process of thick AF/EP laminates. FBG sensors were used to monitor the temperature overshoot phenomenon and the temperature distribution in the thick AF/EP laminate. The highest temperature was noted at the centre of the laminate. Temperature overheating leads to an error of 21.16% in the FBG measurements, as compared with the thermocouple measurements.
2. It was determined that among the three curing factors (heating rate, dwelling temperature, and dwelling time) affecting the curing of the thick AF/EP laminate, dwelling temperature was the primary parameter affecting the overshoot temperature, with a contribution of 75.78% based on the Taguchi method and ANOVA. The contributions of the heating rate and dwelling time were 20.16% and 4.06%, respectively.
3. The relationship between the overheating temperature and curing factors was established using SNR method to predict the overheating temperature. Among the four level combinations of three factors, the optimal curing factors were a heating rate of 0.25 °C/min, dwelling temperature of 80 °C, and dwelling time of 90 min. Compared with the experimental results, the predicted overheating temperature has an error of 2.58%.
4. Compared with the original curing cycle, the overshoot temperature of the optimised curing cycle was reduced by 58.48 °C, representing a reduction ratio of 23.28%.

## Figures and Tables

**Table 1:** L_16_ orthogonal array.

No.	Factor A	Factor B	Factor C
1	1	1	1
2	1	2	2
3	1	3	3
4	1	4	4
5	2	1	2
6	2	2	1
7	2	3	4
8	2	4	3
9	3	1	3
10	3	2	4
11	3	3	1
12	3	4	2
13	4	1	4
14	4	2	3
15	4	3	2
16	4	4	1

**Table 2:** Heating rate, dwelling temperature, and dwelling time of the factors.

Factors	Levels			
	1	2	3	4
Heating rate/°C/min	0.25	0.5	0.75	1
Dwelling temperature/°C	80	90	100	110
Dwelling time/min	30	60	90	120

**Table 3:** Level combinations and experimental values.

No.	Factors			Experimental Results	
	Factor A (Heating Rate/°C/min)	Factor B (Dwelling Temperature/°C)	Factor C (Dwelling Time/min)	Overheating Temperature (°C)	SNR Value (dB)
1	1 (0.25)	1 (80)	1 (30)	206.49	46.30
2	1 (0.25)	2 (90)	2 (60)	217.95	46.77
3	1 (0.25)	3 (100)	3 (90)	222.36	46.94
4	1 (0.25)	4 (110)	4 (120)	222.36	46.94
5	2 (0.5)	1 (80)	2 (60)	218.45	46.79
6	2 (0.5)	2 (90)	1 (30)	224.75	47.03
7	2 (0.5)	3 (100)	4 (120)	244.18	47.75
8	2 (0.5)	4 (110)	3 (90)	249.10	47.93
9	3 (0.75)	1 (80)	3 (90)	206.47	46.30
10	3 (0.75)	2 (90)	4 (120)	232.37	47.32
11	3 (0.75)	3 (100)	1 (30)	239.51	47.59
12	3 (0.75)	4 (110)	2 (60)	239.53	47.59
13	4 (1)	1 (80)	4 (120)	202.93	46.15
14	4 (1)	2 (90)	3 (90)	215.63	46.67
15	4 (1)	3 (100)	2 (60)	247.21	47.86
16	4 (1)	4 (110)	1 (30)	232.56	47.33
				*η_m_*	47.08

**Table 4:** ANOVA of SNR value.

Variables	Level-1	Level-2	Level-3	Level-4	Sum of Squares	Degrees of Freedom	Mean Variance	*F*-Test	Contribution
Heating rate	**46.74**	47.38	47.20	47.00	0.9	3	0.3	4.115	20.16%
Dwelling temperature	**46.38**	46.95	47.54	47.45	3.383	3	1.128	15.464	75.78%
Dwelling time	47.06	47.25	**46.96**	47.04	0.181	3	0.06	0.828	4.06%
Total					4.464				100%

## Data Availability

Not applicable.
